# UPRT, a suicide-gene therapy candidate in higher eukaryotes, is required for *Drosophila* larval growth and normal adult lifespan

**DOI:** 10.1038/srep13176

**Published:** 2015-08-14

**Authors:** Arpan C. Ghosh, MaryJane Shimell, Emma R. Leof, Macy J. Haley, Michael B. O’Connor

**Affiliations:** 1 Department of Genetics, Cell Biology and Development, University of Minnesota, Minneapolis, MN 55455, USA

## Abstract

Uracil phosphoribosyltransferase (UPRT) is a pyrimidine salvage pathway enzyme that catalyzes the conversion of uracil to uridine monophosphate (UMP). The enzyme is highly conserved from prokaryotes to humans and yet phylogenetic evidence suggests that UPRT homologues from higher-eukaryotes, including *Drosophila*, are incapable of binding uracil. Purified human UPRT also do not show any enzymatic activity *in vitro*, making microbial UPRT an attractive candidate for anti-microbial drug development, suicide-gene therapy, and cell-specific mRNA labeling techniques. Nevertheless, the enzymatic site of UPRT remains conserved across the animal kingdom indicating an *in vivo* role for the enzyme. We find that the *Drosophila* UPRT homologue, *krishah* (*kri*), codes for an enzyme that is required for larval growth, pre-pupal/pupal viability and long-term adult lifespan. Our findings suggest that UPRT from all higher eukaryotes is likely enzymatically active *in vivo* and challenges the previous notion that the enzyme is non-essential in higher eukaryotes and cautions against targeting the enzyme for therapeutic purposes. Our findings also suggest that expression of the endogenous UPRT gene will likely cause background incorporation when using microbial UPRT as a cell-specific mRNA labeling reagent in higher eukaryotes.

Pyrimidine nucleosides, apart from being integral components of our genetic code, play important roles in polysaccharide and phospholipid synthesis and detoxification via glucourinidation and glycosylation of proteins and lipids (reviewed in^1^). Thus, biosynthesis of these compounds is crucial for both growth and maintenance of living cells. The uracil nucleotide, uridine monophosphate (UMP), is the precursor to all pyrimidine nucleotides and can be synthesized both *de-novo* from amino acids and via one or more of the salvage pathways that help recycle nucleosides generated by DNA or RNA degradation^1^. In humans and higher eukaryotes, pyrimidine salvage is thought to primarily occur at the nucleoside level where uridine or cytidine is converted back to UMP or CMP by the activity of the enzymes uridine kinase, deoxycytidine kinase and thymidine kinase^1^,^2^. Microbes, however, are capable of salvaging free uracil bases by directly converting uracil to UMP using the microbial enzyme Uracil phosphoribosyltransferase (UPRT) (Fig. 1A)^3^,^4^,^5^,^6^,^7^,^8^. Whether higher eukaryotes, including humans, can salvage uracil for reuse remains unknown with the exception of *Arabidopsis thaliana* which has been shown to have an active UPRT homologue and is therefore capable of salvaging free uracil bases^9^,^10^,^11^.

Homologues of UPRT are found in the genome of all higher eukaryotes, and the gene is reportedly expressed strongly in human blood leukocytes, liver, spleen and thymus and moderately in the prostate, heart, brain, lung and skeletal muscle^10^. However, in contrast to microbial UPRT, purified human UPRT shows no activity *in vitro*^10^. A phylogenetic analysis of UPRT proteins from both microbes and higher eukaryotes reveals that UPRT from higher eukaryotes, including *H. sapiens*, *D. melanogaster* and *C. elegans*, lack two amino acids in the conserved uracil binding site, presumably making enzymes from higher eukaryotes incapable of binding its substrate^10^. Yet, UPRT from almost all species analyzed by Li *et al.* showed complete conservation of the four short regions that encircle the active site of *Toxoplasma gondii* UPRT (*Tg*-UPRT) and are considered essential for enzymatic activity^12^. Additionally, the PRPP binding site of UPRT from higher eukaryotes is also highly conserved across species^10^. These observations indicate that, even though UPRT from higher eukaryotes may not bind uracil, these enzymes may still be active *in vivo* where additional co-enzymes or uracil-binding proteins could present uracil to UPRT for conversion to UMP (also speculated in^10^).

Interestingly, the premise that UPRT from higher eukaryotes is inactive has lead to proposals for both therapeutic and technological uses for microbial UPRT. UPRT is extensively studied in microbes and, since the enzyme is highly active in microbes, UPRT is often seen as a potent drug target for anti-microbial therapy^4^,^5^,^6^. For instance, *Mycobacterium tuberculli*, the causative agent of tuberculosis, has been shown to lack the enzymes uridine nucleosidase, uridine phosphorylase, uridine kinase or uridine monophosphatase which can catalyze conversion of uracil or uridine to UMP^4^,^13^. However, *M. tuberculi* does express an active UPRT enzyme that provides the only route for pyrimidine salvage^6^. Hence, the enzyme is considered essential for survival of the microbe and an ideal target for rational drug design^4^. Use of UPRT has also been proposed for treatment of tumors via suicide-gene therapy, a therapeutic approach involving introduction of a viral or microbial gene into tumor cells and using the gene product to convert a non-toxic compound into a lethal drug^14^,^15^. Microbial Cytosine Deaminase (CD), an enzyme that catalyzes hydrolytic deamination of cytosine to uracil, is a well studied suicide-gene therapy candidate^14^,^16^,^17^. Introduction of the enzyme in tumor cells allows conversion of 5-fluorcytosine (5-FC) to 5-fluorouracil (5-FU), which is toxic to cells when converted by cellular enzymes to potential pyrimidine antimetabolites. One of the cell death mechanisms involves conversion of 5-FU to 5-FUMP that can be incorporated into cellular RNA, and a number of recent studies have shown that introduction of microbial UPRT along with CD can increase the efficiency of the CD/5-FC suicide-gene therapy system^18^,^19^,^20^,^21^.

On the technological front, Cleary *et al.* and Miller *et al.* have shown that in both rodents and *Drosophila*, cell-type specific expression of microbial UPRT can be used to produce labeled cell-type specific RNA (Fig. 1B)^11^,^22^. Ectopic expression of *Toxoplasma gondii*-UPRT in a cell-type of interest was shown to allow cell-specific conversion of a thiolated derivative of uracil, 4-thiouracil (4TU), to 4-thiouridine monophosphate (4TUMP) that is then incorporated into newly synthesized RNA. Since cellular RNA is not thiolated, the thio-labeled RNA can be purified from total RNA by biotinylating the thiol groups and subsequently affinity purifying with streptavidin beads^11^,^22^. While these therapeutic and technological uses of UPRT hold great promise, successful implementation of these techniques will require a deeper understanding of the role of the endogenous UPRT homologues found in mammals and/or model higher eukaryotes.

In this study, we investigated the *in vivo* activity of *Drosophila* UPRT and the role of the enzyme in larval growth and adult survival. We find that the *Drosophila* UPRT homologue Krishah (Kri) is required in both S2 cells and larvae for incorporation of a uracil derivative, 4TU, into cellular RNA indicating that the enzyme is active *in vivo*. Loss-of-function mutations in *kri* led to larval growth defects and lethality that could be rescued by feeding the larvae uridine monophosphate (UMP), the product of the enzymatic reaction that Kri catalyzes or uridine which can be converted to UMP by a number of different kinases. Adults generated by rearing the mutants on UMP showed a significantly shorter life span compared to genetically identical controls that were supplemented with UMP indicating that the activity of the enzyme is also important for maintaining adult physiology. Taken together these observations indicate that a UPRT homologue from *Drosophila melanogaster*, a higher eukaryote, is active *in vivo* and suggests, contrary to current views, that UPRTs from other higher eukaryotes are also likely to have *in vivo* enzymatic activity that have consequences for both therapeutic and technological uses of this enzyme.

## Results

### *Drosophila* UPRT homologue, *krishah (kri)*, encodes for an active enzyme in S2 cells

**Figure 1:**
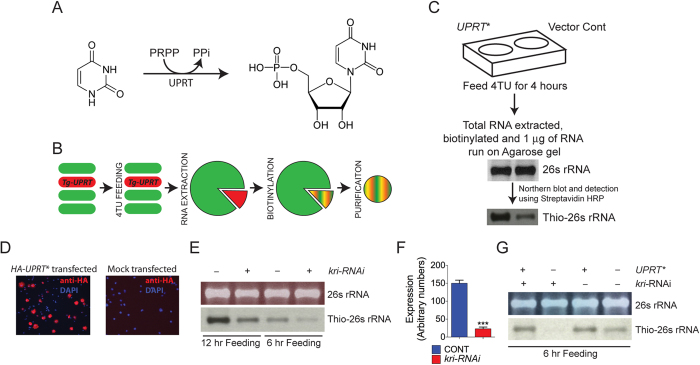
*Drosophila* UPRT is active in S2 cells. (**A**) UPRT catalyzes the conversion of uracil to uridine monophosphate in the presence of phosphoribosyl pyrophosphate (PRPP). (**B**) A schematic of the 4TU-tagging technique proposed for cell-type specific labeling of RNA. (**C**) Work flow of 4TU labeling in S2 cells and detection of 4TU labeled RNA using a Northern blot assay. S2 cells transfected with UPRT* show robust labeling of RNA with 4TU. Mock-transfected cells also show significant incorporation of 4TU in cellular RNA. Total RNA loaded in each lane can be seen in the agarose gel picture. (**D**) Anti-HA staining of S2 cells transfected with HA-UPRT* or vector alone shows the specific presence of the HA-UPRT* protein in test cells only. (**E**) 4TU incorporation in RNA extracted from S2 cell that were treated with or without ds-kri-RNA (*kri-RNAi*). *kri-RNAi* strongly suppresses 4TU labeling of 26S RNA with both 6 and 12 hrs of 4TU feeding. (**F**) Quantitative-PCR verifies a significant reduction in *kri* expression in S2 cells treated with *kri-RNAi*. (**G**) Transfection with UPRT* can rescue 4TU incorporation in *kri-RNAi* cells. For all northern blots 2 μg of total RNA was loaded per lane. Ethidium bromide (EtBr) staining of agarose gels is shown in the upper panel of each northern blot figure. Gell images and blots shown here are cropped images of larger files and full-length blots/gels are shown in Supplementary Figure S3. All blots shown in this figure were exposed for 30 seconds. Longer exposures of the blots are shown in Supplementary Figure S4.

Our original goal was to make a *Tg-UPRT* (UPRT homologue from *Toxoplasma gondii*) construct that is codon optimized for *Drosophila* (that is referred hereafter as UPRT*) so that it could be used for high efficiency cell-type specific RNA labeling in *Drosophila*. To test the enzymatic activity of UPRT*, we transiently expressed UPRT* in *Drosophila* S2 cells. UPRT* transfected and control cells (transfected with vector alone) were fed 4TU and cellular RNA was extracted from these cells. Northern blots probing for the presence of 4TU in RNA revealed robust incorporation of 4TU in UPRT* transfected cells (Fig. 1C,D). Interestingly, we saw significant incorporation of 4TU in control S2 cells, indicating the presence of an endogenous salvage pathway that can incorporate 4TU. The *Drosophila* genome contains a single un-annotated *UPRT* homologue identified as *CG5537*. In accordance with the convention of naming *Drosophila* genes based on phenotypes observed in mutants (see below) we name this gene *krishah* (*kri*). We hypothesized that incorporation of 4TU in S2 cells transfected with vector alone is caused by the activity of Kri. To test this hypothesis, we knocked-down *kri* in S2 cells and asked if incorporation of 4TU is affected. Knocking down *kri* in S2 cells by addition of *kri*-dsRNA led to a strong decrease in 4TU incorporation in S2 cells both when the cells were fed for 12 hours or 6 hours with media containing 4TU (Fig. 1E). We used quantitative PCR to verify the efficiency of *kri*-*RNAi* and found a ~6.5 fold decrease in UPRT expression in the RNAi treated S2 cells vs. the non-treated S2 cells (Fig. 1F). To further verify that the loss of 4TU incorporation is not due to an off target effect, we tried rescuing the loss of 4TU incorporation in presence of *kri-RNAi* by transfecting the cells with UPRT*. The UPRT* sequence is highly divergent from *kri* and *kri*-*RNAi* is not expected to affect UPRT* mRNA levels. Consistently, we found that transfection with UPRT* could restore 4TU incorporation even in presence of *kri*-*RNAi* while non-transfected cells showed negligible 4TU incorporation (Fig. 1G). These results show that the *Drosophila* UPRT homologue Kri is involved in 4TU incorporation into cellular RNA, indicating that Kri can actively convert 4TU to 4TUMP *in vivo* in S2 cells.

### Activity of *kri* in *Drosophila* larvae and generation of *kri* mutants

**Figure 2:**
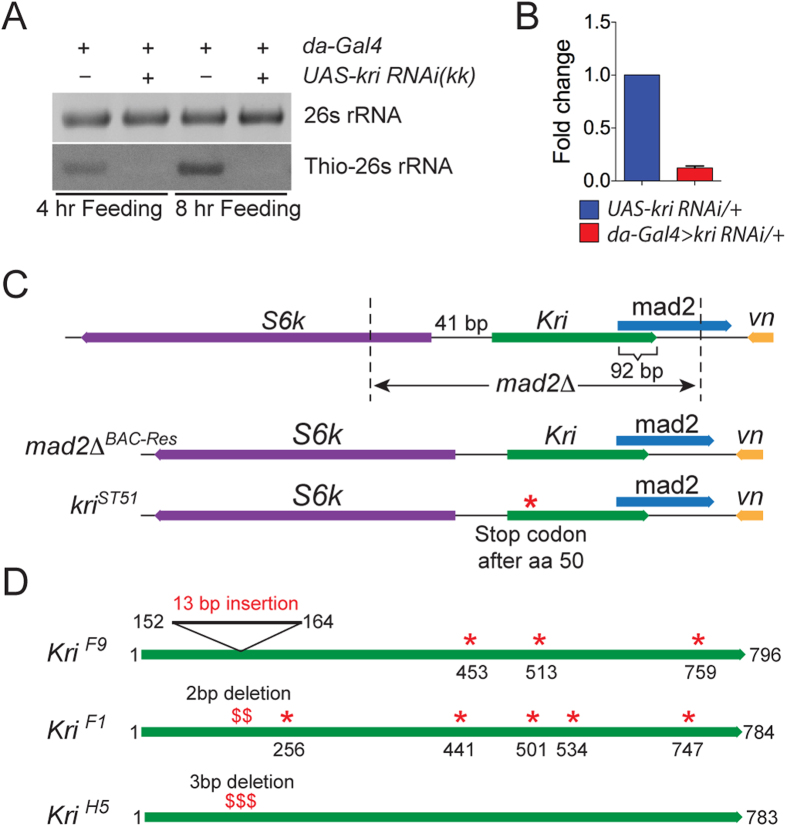
*Drosophila* UPRT is active in the larvae. (**A**) 4TU incorporation in 26S RNA of third instar Gal4-control and *kri-RNAi* larvae that were fed with 4TU for 4 hrs and 8 hrs. 2 μg of total RNA was loaded per lane and EtBr staining of agaraose gel is shown in upper panel. Gel image and blot shown here are cropped images of larger files and full-length blots/gels are shown in Supplementary Figure S3. The blot shown here was exposed for 30 seconds. (**B**) Verification of *kri* knockdown in *kri-RNAi* larvae using qPCR show a ~10 fold decrease in *kri* mRNA level. (**C**) Schematic of the selective gene replacement strategy used for generating *mad2*Δ^*BAC-Res*^ and *kri*^*ST51*^ mutant flies. (**D**) Lesions in the *kri*^*F9*^, *kri*^*F1*^ and *kri*^*H5*^ alleles generated using CRISPR. Red asterisks represent stop codons.

To determine if Kri is active in the organism, we examined 4TU incorporation in *Drosophila* larvae. We chose to look at the larval stage since *Drosophila* larvae grow at a remarkably fast rate and therefore we reasoned that the uracil salvage pathway might be necessary for maintaining this fast growth rate. *Drosophila* larvae were grown in axenic conditions for 4TU incorporation assays to avoid any possible facilitation of 4TU incorporation by commensal gut microbes or microbes present in the food. We found that control third instar larvae were able to incorporate 4TU in cellular RNA when fed for either 4 or 8 hours with 4TU supplemented food (Fig. 2A). However, under identical conditions, larvae expressing a *kri-RNAi* (VDRC, *kk 107789*) construct under the regulation of a pan larval driver (*da-Gal4*) showed strong reduction in incorporation of 4TU (Figs 2A and 5B,C). Quantitative PCR with whole larval RNA extracts confirmed the efficiency of the *kri* RNAi line used in these experiments (Fig. 2B). These results show that Kri is active in *Drosophila* larvae and can lead to incorporation of a uracil derivative, 4TU, into cellular RNA. To determine the tissue distribution of *kri* mRNA we carried out *in situ* hybridization experiments in embryos and larvae (Supplement Fig. S1). Consistent with previously reported tissue and stage-specific RNA-Seq expression profiles (flybase), we find that *kri* is ubiquitously expressed, perhaps indicating that all cells have the ability to salvage uracil via this pathway during development.

**Figure 3:**
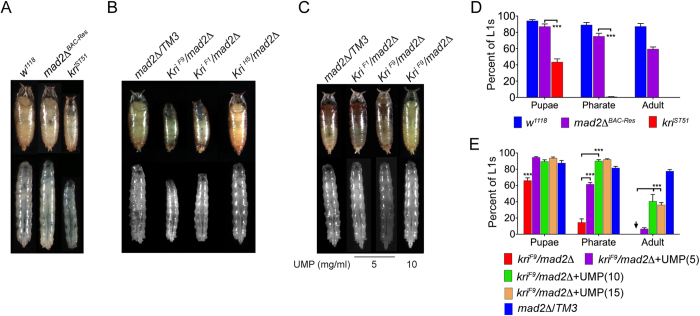
*kri* is essential for larval growth and larval/pupal viability. (**A**) *w*^*1118*^control and *kri*^*ST51*^ mutant wandering third instar larvae and pupae. Animals were grown on standard CMF. (**B**) *kri*^*F9*^*/mad2*Δ and *kri*^*F1*^*/mad2*Δ mutant and control wandering third instar larvae and pupae. Animals were grown on standard CMF. (**C**) *kri*^*F9*^*/mad2*Δ and *kri*^*F1*^*/mad2*Δ mutant and control wandering third instar larvae and pupae that were grown on CMF supplemented with 5 mg/ml UMP. Last lane shows *kri*^*F9*^*/mad2*Δ mutants that were grown on CMF supplemented with 10 mg/ml UMP. (**D**) Larval and pupal viability of *w*^*1118*^ and *mad2*Δ^*Res*^ controls and *kri*^*ST51*^ mutants presented as percent of L1s that progress to pupal, pharate or adult stages (*n* = 6 groups of 40 L1s each). (**E**) Larval and pupal viability of *kri*^*F9*^*/mad2*Δ mutants reared on either CMF alone or CMF supplemented with 5 mg/ml, 10 mg/ml or 15 mg/ml UMP. *mad2*Δ/*TM3* controls were raised on CMF + 5 mg/ml UMP (*n *= 6 groups of 60 L1s each). All quantitative data presented as mean ± SEM.

To determine if Kri activity is necessary for *Drosophila* viability, we produce loss of function phenotype via RNAi knockdown and gene mutation. While one of the two *kri-RNAi* lines (*kk 107789*) available from VDRC showed a reduction in 4TU incorporation, we observed no growth defects when ubiquitously expressed. In contrast, expression of the other line (GD 22158) using *da-Gal4* driver led to larval growth defects indicating that *kri* mediated salvage of uracil may be necessary to maintain the vigorous growth manifested by *Drosophila* larvae. To test this hypothesis, we generated *kri* mutants and looked for larval growth defects. The *kri* gene codes for a 1.1 kb transcript that is located 41 bp upstream of the *Drosophila S6k* coding region. Since *S6k* is an essential gene, we could not employ standard P-element excision to generate *kri* mutants, as these might be expected to affect *S6k* expression. Instead we used two independent strategies to generate flies that lacked an active Kri protein and yet had normal *S6k* expression. First, we employed a selective gene replacement strategy to generate flies lacking *kri*. We started with a previously characterized deletion mutant called *mad2*Δ that removes all of the *kri* coding region, the last 180 bp of a downstream gene *mad2* and first 1120 bp of *S6k* (See Fig. 2C for a schematic). We can rescue the lethality associated with the deletion by introducing a 22 kb BAC (CH322-05D15), that includes all the three genes, on the second chromosome of the *mad2*Δ flies (Fig. 2C ‘*mad2*Δ^*BAC-Res*^’ and Fig. 3A). To generate *kri* mutants, we used recombineering to introduce a stop codon in the coding region of *kri* in the BAC and used this BAC to rescue *mad2*Δ flies (Fig. 2C ‘*kri*^*ST51*^’). The recombineered BAC thus rescues for *S6k* and *mad2* deficiency but not for *kri*. During the course of this study, CRISPR-mediated mutagenesis tools became available, and we also used CRISPR-mediated NHEJ as a second strategy to introduce point mutations in the genomic *kri* locus^23^,^24^. We generated two independent *kri* mutants using CRISPR, 1) *kri*^*F1*^ which is a 2 bp frame shift mutation at the targeted region and leads to 4 stop codons downstream, 2) *kri*^*F9*^ which is a 13 bp insertion at the targeted site and leads to 3 stop codons downstream (Fig. 2D). In both cases stop codons were introduced well before the catalytic domain of Kri, and therefore, both mutants are expected to produce an inactive enzyme. We also retrieved a mutant, *kri*^*H5*^, which has an in-frame 3 bp deletion at the targeted site (Fig. 2D). We used the *kri*^*H5*^line as a control for some of our experiments.

### Kri activity is required for larval growth and pupal viability

The *mad2*Δ deletion leads to lethality in the early stages of larval development, and these mutants never reach the wandering larval stage. Introduction of a single copy of the BAC CH322-05D15 into these flies completely rescues lethality producing adults and the rescued wandering third instar larvae and pupae were indistinguishable from the *w*^*1118*^ control (Fig. 3A,D). However, a single copy of the recombineered BAC that contains a stop codon in the *kri* coding region leads to a significant increase in larval lethality (Fig. 3D). Nevertheless, a large number of these animals do reach the wandering larval stage and form pupae/pre-pupae (Fig. 3D). Interestingly, wandering mutant larvae and pupae are much smaller and thinner than controls indicating larval growth defects. The mutant pupae rarely make it to the pharate stage and never eclose as adults. Based on the thin-larva phenotype of the mutants we named the gene *krishah*, which in Sanskrit means thin. The same larval growth defect was also observed in the *kri* mutants generated using CRISPR. Two independent *kri* alleles, when heterozygosed with the *mad2Δ* deletion, lead to formation of thin third instar larvae and pupae/pre-pupae (Fig. 3B). Similar to the *mad2*Δ^*BAC-St51*^ mutants, *kri*^*F9*^*/mad2*Δ and *kri*^*F1*^*/mad2*Δ animals also failed to reach the pharate stage in most cases and never eclosed as adults (Fig. 3E). However, both the *kri*^*F9*^ and *kri*^*F1*^ alleles were homozygous lethal as embryos or early first instars indicating potential CRISPR-induced secondary mutations. To ensure that the 2 bp deletion in the *kri*^*F9*^ allele does not lead to the thin larva phenotype by affecting *S6k* expression, we examined the *kri*^*H5*^*/mad2Δ* mutants since *kri*^*H5*^ is an in-frame 3 bp deletion in the same location as *kri*^*F9*^. The *kri*^*H5*^*/mad2Δ* animals do not show any larval growth defect or pupal lethality (Fig. 3B). Additionally, both the *kri*^*F1*^ and *kri*^*H5*^ alleles complement *S6k*^*07084*^, a lethal *S6k* allele, confirming that these mutations do not significantly affect *S6k* function and that the thin-larval phenotype is specific for loss of *kri* (Table 1).

To confirm that the enzymatic activity of Kri is indeed needed for larval growth, we examined whether we could rescue the larval growth defects and pupal lethality by feeding the mutants food supplemented with UMP. Since UMP is highly soluble in aqueous media and, unlike purine nucleotides, is known to be absorbed across the intestinal epithelia in mammals, we presumed that larvae would be able to absorb this compound. Indeed, rearing the *kri* mutants on food containing as little as 5 mg/ml of UMP significantly rescued larval growth and larval/pupal viability (Fig. 3C,E). While only a small fraction of the mutants supplemented with 5 mg/ml UMP eclosed as adults, rearing these animals on 10 or 15 mg/ml UMP resulted in 40–50% of the mutants eclosing as adults (Fig. 3E). Notably, most of the mutant adults that eclosed on 10 or 15 mg/ml of UMP were males presumably because they are smaller in size and residual larval growth retardation leading to enhanced pupal lethality for the mutant females. We also tested whether *kri* mutants could be rescued by uridine, which can be converted to UMP by uridine kinase. Uridine is also able to rescue *kri* mutants as well on a per mole basis as UMP at low concentration (Fig. S2). However since it is significantly less soluble in aqueous media than UMP, this concentration was not sufficient to obtain adult rescue. In summary, two independent strategies used to generate loss-of-function mutations in *kri* lead to strong larval growth defects and significant larval/pupal lethality that can all be rescued by feeding the mutants UMP or uridine. These results strongly suggest that Kri-mediated salvage of uracil is essential for maintaining the fast growth observed in *Drosophila* larvae.

### Kri is required for viability of adult flies

**Figure 4:**
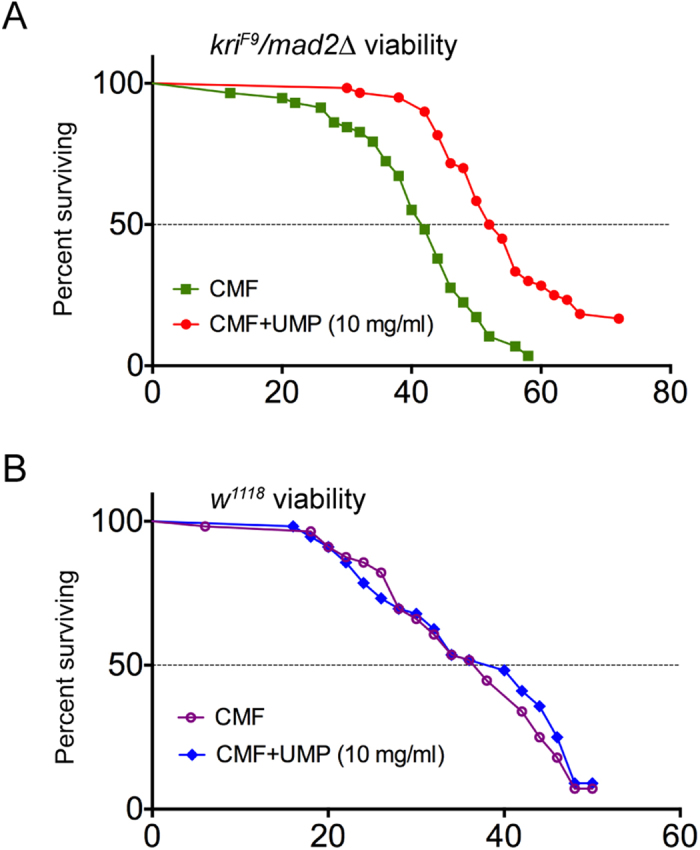
*kri* is required for maintenance of adult viability. (**A**) Viability of *kri*^*F9*^*/mad2*Δ adults on CMF and CMF supplemented with 10 mg/ml of UMP. Viability of the mutants on standard CMF was significantly lower (p < 0.0001) as determined by the Kaplan-Meier log rank test. (**B**) Viability of *w*^*1118*^ adults on CMF and CMF supplemented with 10 mg/ml of UMP. *n *= 60 flies per genotype per condition.

While salvaging of uracil via Kri may be essential to maintain larval growth, the enzyme could be dispensable for viability of adult flies. To test if *de novo* synthesis of UMP is sufficient to maintain adult viability, we tested the effect of feeding UMP on the viability of *kri* mutant flies. Adult *kri* mutant males were obtained by rearing larvae on cornmeal food (CMF) supplemented with 10 mg/ml UMP. These flies were divided into two groups of 60 flies each and maintained on either CMF (control.) or CMF +10 mg/ml UMP (test) to determine adult life span. Since the test and control flies were of identical genotype and were grown under identical food and rearing conditions, any difference in life span should be purely caused by the availability or deficiency of UMP. We found that *kri*^*F9*^*/mad2*Δ flies lived significantly longer when maintained on CMF supplemented with UMP indicating that Kri-mediated salvage of uracil is necessary for maintaining healthy adult flies (Fig. 4A). The presence of UMP however, did not have any effect on the lifespan of *w*^*1118*^ isogenic flies suggesting that UMP by itself does not affect *Drosophila* adult life span and that the differences in lifespan observed in *kri* mutants is due to lack of Kri activity (Fig. 1B). We observed that *w*^*1118*^ flies had a much shorter lifespan compared to even *kri* mutants maintained on CMF alone. This difference is likely due to differences in genetic background, *w*^*1118*^ being isogenic (VDRC 60000) and *kri*^*F9*^*/mad2*Δ flies being heterozygous for all chromosomes. Additionally, differences in rearing conditions of the *kri* (reared at very low densities of 60 larvae per fly) mutant and *w*^*1118*^ (reared in bottles at moderate density) larvae also affected the adult life span in these independent experiments.

### A *Drosophila* codon optimized tgUPRT* line with high efficiency incorporation of 4TU

**Figure 5:**
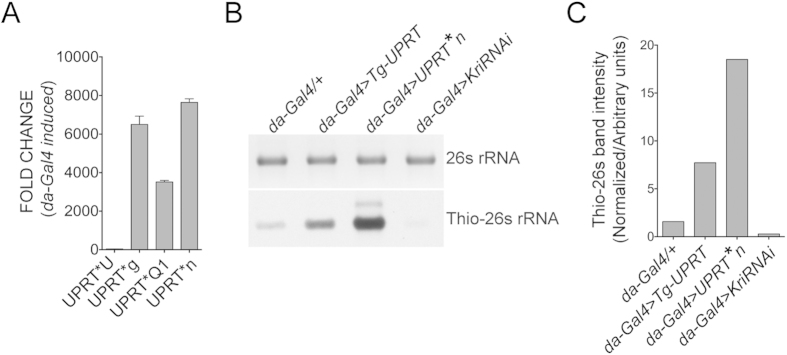
UPRT*: A codon optimized *Tg-UPRT* line for more efficient incorporation of 4TU. (**A**) Relative expression level of four different UPRT* lines in *Drosophila* using the ubiquitous *da>Gal4* driver. (**B**) Relative incorporation levels of 4TU into RNA in mid third instar larvae of the genotypes *da-Gal4/+* (lane1), *da-Gal4*>*Tg-UPRT* (lane2), da-Gal4>UPRT*n (lane3) and *da-Gal4>kriRNAikk* (lane4). (**C**) Densitometric quantification of thiolated 26S RNA bands in B normalized to loading control. The gel image and blot shown here are cropped images of larger files and full-length blot/gel are shown in Supplementary Figure S3. The blot shown here was exposed for 5 seconds. Longer exposure of the blot is shown in Supplementary Figure S4.

As described, our initial goal was to try to use codon optimization to improve efficiency of *in vivo* 4TU labeling in *Drosophila*. We generated four independent transgenic lines that express UPRT* under the control of a UAS promoter. We tested each of these lines for inducibility by the pan larval driver *da-Gal4*. Three of the four lines show strong induction of UPRT* expression in presence of Gal4 (Fig. 5A). All four lines show negligible amount of leaky expression in absence of Gal4. We checked one of these lines (UPRT*n) for its ability to incorporate 4TU and found a 12 fold increase of 4TU labeling over endogenous UPRT activity and a 2.5 fold enhancement relative to the available non-optimized *Tg-UPRT* line (Fig. 5B,C). Since we see significant variation in the *da-Gal4* mediated induction of different UPRT* lines, it is possible that the improvement in 4TU labeling efficiency is just a reflection of insertion site effects. Nevertheless, when induced by the same *Gal4* driver, the UPRTn* line shows much improved 4TU incorporation in comparison to the currently available *Tg-UPRT* line and should prove valuable for researchers wishing to utilize *in vivo* 4TU labeling in *Drosophila*.

## Discussion

In mammals and other higher eukaryotes pyrimidine salvage is known to occur at the nucleoside stage^1^,^2^. Alternatively, a small fraction of uracil could also be salvaged by the reversible activity of the uridine phosphorylase enzyme in mammals thereby allowing a two-step conversion of uracil to uridine and then to UMP. Interestingly, all higher eukaryote genomes code for a highly-conserved homologue of the enzyme uracil phophoribosyltransferase (UPRT) that, in microbes, can directly convert uracil to UMP. However, the ability of this enzyme to actually catalyze the conversion of uracil to UMP in mammals and higher eukaryotes remains unproven^10^,^11^. We report that *Drosophila* UPRT (Krishah) is required both in cultured S2 cells and in the larvae for incorporation of a uracil homologue (4TU) into cellular RNA, a process that requires conversion of 4TU to 4TUMP. Therefore, we conclude that Kri is capable of recognizing 4TU as a substrate *in vivo* and converting it to 4TUMP and can likely also convert uracil to UMP. Kri shares strong sequence similarity with UPRT homologues from other higher eukaryotes including humans^10^. The four short regions of the UPRT family that form the active catalytic site in *Tg*-UPRT are completely conserved in Kri, much like the human UPRT, and the PRPP binding site is also identical to other UPRT homologues^10^. Interestingly, Kri also has a uracil-binding region that is similar to human-UPRT and is missing two conserved glycines that are considered essential for substrate recognition from studies on microbial UPRTs^10^. This lack of the two conserved glycines has been proposed to be the reason why purified human-UPRT does not show any activity *in vitro*^10^. However, the fact that Kri can recognize a uracil derivative and catalyze its conversion to a ribonucleoside monophosphorylated form argues that human-UPRT should also be capable of binding uracil *in vivo* and converting it to UMP. This could be accomplished via either a cofactor that binds uracil and brings it to the UPRT enzyme, or by a yet uncharacterized alternative uracil-binding site contained in the UPRT homologues from higher eukaryotes.

In most organisms pyrimidine salvage predominates over *de novo* synthesis of these nucleotides for maintaining cellular growth and proliferation. As such, the importance of the pyrimidine salvage pathway enzymes become even more pronounced under scenarios that involve rapid growth of a tissue or an organism (especially uracil salvage, as it is required for maintaining high turn-over of mRNAs). Consistent with this notion, we find that loss of *kri* leads to severe growth defects in *Drosophila* larvae that are known to undergo a massive 100–150 fold increase in size during the four days of the larval life. *kri* mutant larvae that survive to the pupal stage die as prepuae and pharates most likely because the animals failed to gain enough body mass to sustain metamorphosis. When these mutants are grown on food supplemented with UMP or Uridine, larval growth was significantly rescued and so was viability at the pupal stage and eclosion of viable adults when fed UMP. Interestingly, while larval lethality is completely rescued on UMP supplemented food (percent of L1s that formed pupae, Fig. 3E), almost all of the mutant adults that eclosed on UMP supplemented food were males (females dying in pupal or pharate stage). This finding further supports the idea that the pupal lethality of the *kri* mutants is associated with the mass of the larvae when they enter metamorphosis. Adult *Drosophila* males are significantly smaller compared to females, and growth-attenuated male larvae have a higher chance of eclosing as adults compared to females. While the necessity of a robust pyrimidine salvage pathway to sustain the fast growth associated with the larval stage is understandable, it was interesting to see that Kri is also required for maintaining the adult lifespan. We do not know how Kri affects adult life span and the mechanism(s) could involve the requirement of uracil salvage in mRNA production to maintain cellular physiology, maintenance of stem cell proliferation and/or yet unknown role(s) of the protein. Overall, our findings provide strong evidence supporting an *in vivo* role of the *Drosophila* UPRT homologue, Kri, in uracil salvage, larval growth and adult lifespan and suggest similar roles for UPRT homologues from other higher eukaryotes.

Our findings also have implications for use of UPRT as a cell-type specific labeling reagent^11^,^22^. Clearly endogenous Kri contributes to background incorporation of thio-labeled substrates (Figs 1, 2 and 5). We suggest that additional genetic manipulation may enable this method to produce cleaner cell-type specific labeling. For example, in *Drosophila* it may be possible to rescue the growth defects associated with loss of the endogenous UPRT gene by expressing *kri* in only one tissue. This would allow for expression of the exogenous optimized UPRT* gene in other tissues and thereby improve the sensitivity of the method. We do not rule out the possibility of optimizing 4TU feeding-time or concentration of 4TU in the food to maximize the signal to noise ratio for achieving tissue specific labeling. However, if this approach is chosen, we recommend the use of the new codon optimized UPRT* lines as they can facilitate stronger incorporation of 4TU and thereby further improve the signal to noise ratio. Alternatively, one could look for uracil derivatives that are not recognized by *Drosophila* UPRT and yet can act as substrates for an optimized microbial UPRT allowing tissue specific labeling.

## Materials and Methods

### Fly strains

*UAS-KriRNAi* lines were obtained from VDRC (KK 107789 and GD 22158), *mad2*∆ flies were a generous gift from Dr. Roger Karess lab^25^, isogenic *w*^*1118*^ flies were obtained from VDRC (60000). The non-codon optimized *UAS-tgUPRT* line was obtained from the Bloomington stock center (# 27604) and is described in Miller *et al.*^22^. *kri*^*F1*^, *kri*^*F9*^ and *kri*^*H5*^ lines were generated using CRISPR mediated non-homologuous end joining using a single guide RNA. Mutants were screened using a High-resolution melt assay that employed a Roche quantitative PCR machine as described elsewhere^26^,^27^. *mad2∆*^*BAC-Res*^ and *kri*^*St51*^ flies were generated by integrating BAC-CH322-05D15, and BAC-CH322-05D15 containing a stop codon, respectively into the *attP* docking site located on the chromosome arm 2L of the VK37 fly line^28^ (fly injections were done by BestGene Inc.). pU6-BbsI-chiRNA plasmid for cloning and expression of gRNAs used for CRISPR was obtained from Addgene (45946).

### *in situ* Hybridization

Digoxigenin (Roche) labeled RNA was prepared from cDNA LD21741, using EcoRI digestion and Sp6 polymerase transcription to make the anti-sense probe and SspI digestion and T7 polymerase transcription to make the sense probe. The probes were diluted 1:30, and anti-digoxigenin-AP was diluted 1:5000.

### Use of BAC recombineering to introduce stop codon in *kri*

We started with a CHORI clone (28, BacPac Resources), CH322-05H15, that spans the *mad2, CG5537 (kri)* and *s6k* genes. The recombineering protocol described in Venken *et al.*^29^ was followed with a variation in primer design. Instead of using the N-EGFP-F primer, the forward primer contained *kri* sequence up to the T in codons 20 (Ser TCA) or 51 (Ser TCG) fused to the GA of codons 230G and 231I at the end of GFP prior to *loxP*, resulting in GFP being out-of-frame. The reverse primer was the usual N-term-PL452-R^29^ fused to homologous *kri* sequence past the desired stop, and the tag template vector was PL-452 N-EGFP (Addgene #19173). Heat shock-induced SW102 cells containing CH322-05H15 were electroporated with the PCR selection cassette and selected for Kan resistance. Recombinant BACs were then electroporated into arabinose-induced SW106 to obtain the final BAC with a stop codon in *kri.* The recombineered sequence was confirmed to be: *kri*—stop and out-of-frame EGFP—stop—*loxP*—stop—vector sequence and out-of-frame *kri* sequence.

### Recombineering Primers

UPRTstop20for (*kri* sequence underlined, PL-452 N-EGFP sequence in bold)

CGGCTCGCCCAGCAGCAGCGGCTCCCAGTCGGAGGAGGGCAGCAGTTCCT**GATCACTCTCGGCATGGACGAGCTG**

UPRTstop20rev (*kri* sequence underlined, PL-452 N-EGFP sequence in bold)

CAGCTGTTGCTCCTGCTCCTGGGCTTCCTGCTGCGGCTGCTGGTGATCTG**ACTAGTGGATCCCCTCGAGGGAC**

UPRTstop51for (*kri* sequence underlined, PL-452 N-EGFP sequence in bold)

ACAGCTGCACACTCCCACGCATGCACATGCCGCGGTGCCAGCTGCCACAT**GATCACTCTCGGCATGGACGAGCTG**

UPRTstop51rev (*kri* sequence underlined, PL-452 N-EGFP sequence in bold)

GCACTCGAGTAGCTTCAGGTTGCTGCCGTACTCCGCCAGGATCTCCTCCG**ACTAGTGGATCCCCTCGAGGGAC**

### Primers (for PCR analysis)

UPRT03F    CCAATTGCTAGAAATTACCGAGG

UPRT04R    GAGACGGTCGGCATAGAAC

### Primers (for sequencing)

UPRT01F    GTAGCCAGTGATAGCAACCAAATAG

UPRT02    RCGCAATATGGTCAGCAGTTCGG

### CRISPR mediated Mutagenesis

CRISPR mediated genome-editing methods described in http://flycrispr.molbio.wisc.edu/protocols (FlyCRISPR) website was followed for generating the NHEL mediated mutations in *kri.* gRNA targeting the *kri* gene was designed to introduce mutations early in the kri coding region so that stop codons are introduced before the conserved regions of the gene.

gRNA sequence: GGTGCCAGCTGCCACATCGG

gRNA was cloned in the pU6-BbsI-chiRNA plasmid (Addgene 45946) using BbsI. Fly injection was performed by BestGene Inc.

### Viability assays

For *Drosophila* larval and pupal viability assays 150 virgin females and 120 males of appropriate genotype were mated in an egg-laying cage. On day three after mating was started eggs were collected on an apple juice plate for a short period of 3 hours. 24 hours later 1^st^ instar larvae of appropriate genotype (identified by looking for balancer chromosomes containing fluorescent markers) were transferred to food vials in groups of 60 per vial. Larval viability was calculated as the percent of L1s that reached pupal stage. Pupal viability was measured by calculating the percent of L1s that eclosed as adults. For adult viability assays 60 male flies that eclosed within a period of 24 hours were transferred to appropriate food vials. The flies were flipped every 2 days into a new food vial and the number of dead flies was counted. Survival distribution of the test and control conditions were compared using the Kaplan-Meier log rank test in Graphpad Prism.

### S2 cell transfection

We passaged S2 cells 4 days before transfection. On day 4 we harvested S2 cells and plated at a density of 2 × 10^6^ cells/ml in serum free Schneider’s media (SFM). Transfection was carried out using cellfectin II transfection reagent. Briefly, in parallel two aliquots of 120 μl SFM was mixed with 10 μl cellfectinII reagent and 1.2 μg of DNA respectively. The mixtures were incubated at RT for 15 mins. At the end of the incubation period the mixtures were mixed together and incubated at RT for another 10–15 min before adding to S2 cells in a drop wise fashion. 24 hours post transfection the media from the culture wells were carefully removed and replaced with fresh Schneider’s media containing 10% fetal calf serum (FCS). 4TU feeding was performed 4 days after transfection.

### S2 cell RNAi

S2 cells were plated in 1.4 ml of M3 Shields and Sang media containing insect medium supplement (Sigma) at a density of 2 × 10^6^ cells/ml in 6 well cell culture plates. Two days after plating S2 cell media was replaced with 1.4 ml fresh SFM containing 10 μg of ds-RNA per well. The process was repeated 2 days after the first RNAi treatment and 4TU feeding was performed 3 days after the second RNAi treatment to ensure strong knockdown of the target gene.

### 4TU uracil feeding of S2 cells

4TU feeding was performed in Schneider’s media supplemented with 2% FCS. A 200 mM stock of 4TU in DMSO was diluted 400 times to a final concentration of 0.5 mM for the feeding experiments. S2 cell media was carefully removed without disturbing the cells at the bottom of the plate and 4TU-containing media was gently added to the wells (1.4 ml per well). Feeding was performed for either 6 hours or 12 hours at the end of which S2 cells were harvested by gently spinning them at RT. Cells were washed twice in PBS before RNA extraction.

### 4TU feeding of larvae

Larvae were grown in axenic conditions to avoid absorption of 4-thio-UMP produced by gut microbes (see below) as UMP is known to be readily absorbed across intestinal membranes in mammals^1^. Mid third instar larvae were floated with filter sterilized 20% sucrose and transferred to sterile CMF supplemented with 0.5 mM 4TU. Feeding was performed at 25 ^°^C for 4 hours or 8 hours and at the end of the feeding period larvae were again floated with 20% sucrose, washed three times in cold PBS and were used for RNA extraction.

### Generation of axenic larvae

Embryos were collected over a period of 5–6 hours on apple juice plates. Subsequently they were dechorionated for 2 min with 50% household bleach and washed briefly in sterile water. Embryos were then washed twice in 70% ethanol and then twice with sterile water before transferring to vials of autoclaved CMF (Bloomington recipe). CMF was autoclaved at 120 psi for 15 mins with a quick release of steam to avoid prolonged exposure to heat and charring of sugars.

### Uridine and UMP feeding

Uridine and UMP were added to cornmeal food (brewer’s yeast) in vials atat various concentrations as indicated in figures. Thirty to forty *kriF9/mad2∆* first instar larvae were seeded onto CMF vials containing uridine, UMP, or no supplementation. Larvae were allowed develop at 25 °C and each vial scored for number of pupae, pharates, male and female adults.

### Northern blots for detection of thiolated-RNA

1 μg of total RNA was diluted to a total volume of 5 μl and mixed with 5 μl of RNA loading dye containing ethidium bromide (Invitrogen). The sample was heat treated for 10 minutes at 70 ^o^C and chilled immediately on ice and then centrifuged briefly before loading on 2% formaldehyde and 1% agarose gels (running buffer: 20 mM MOPS, 8 mM C_2_H_3_NaO_2_, 0.5 mM EDTA, pH 7) for separation. RNA was subsequently transferred overnight to a positively charged nylon membrane using a standard Northern blot protocol. Post transfer the blot was UV cross-linked before processing for streptavidin-HRP probing. For streptavidin-HRP probing nylon membranes were first blocked in 10X blocking solution (125 mM NaCl, 17 mM Na_2_HPO_4_, 7.3 mM NaH_2_PO_4_ and 1% SDS) for 20 min at RT. Subsequently the membrane was incubated with 0.66 μg/ml (1/1500 dilution of a 1 mg/ml stock of Streptavidin-HRP, Pierce) Streptavidin-HRP in 10X blocking solution for 5 mins at RT, washed twice with 1X blocking solution for 10 mins each and finally washed twice with 1X assay buffer (10 mM Tris, 10 mM NaCl, 1 mM MgCl_2_ and pH adjusted to 9.5) for 10 mins each. The blot was then treated with ECL reagent and exposed to an X-ray film using standard ECL protocols.

## Additional Information

**How to cite this article**: Ghosh, A. C. *et al.* UPRT, a suicide-gene therapy candidate in higher eukaryotes, is required for *Drosophila* larval growth and normal adult lifespan. *Sci. Rep.* **5**, 13176; doi: 10.1038/srep13176 (2015).

## Supplementary Material

Supplementary Information

## Figures and Tables

**Table 1:** Complementation test between *kri* mutants and an *S6k* mutant.

Crosses	Observed #			Observed %			Total Flies
	Ser^+ve^	Sb^+ve^	Ser−^ve^+Sb^−ve^	Ser^+ve^	Sb^+ve^	*Ser*^*−ve*^*+Sb*^*−ve*^	
*Kri*^*F1*^*/TM3Ser X S6k*^*07084*^*/TM3Sb*	156	130	185	33.1	27.6	39.2	471
*Kri*^*F1*^*/TM3Ser X S6k*^*07084*^*/TM3Sb*	232	210	262	32.9	29.8	37.2	704

Both *kri*^*F9*^ and *kri*^*F1*^ alleles can complement a lethal *S6k* mutation showing that lethality associated with *kri* mutants is not due to the effect of the mutations on the *S6k* promoter. The expected percentages for the three genotypes recognized as *Ser*^*+ve*^, *Sb*^*+ve*^ and *Ser*^*−ve*^*+Sb*^*−ve*^ flies is 33.33% for each cross.

Calculated χ^2^ values: *kri*^*F9*^ = 0.061, *kri*^*F1*^ = 0.025.

χ^2^_0.95_ (*p < 0.05*) for 2 degrees of freedom = 0.103.
