# Supplementary material for: UPRT, a suicide-gene therapy candidate in higher eukaryotes, is required for Drosophila larval growth and normal adult lifespan
Source: Sci Rep. 2015 Aug 14;5:13176. doi: 10.1038/srep13176 (PMC4536494; doi:10.1038/srep13176)
Supplement: Supplementary Information [file srep13176-s1.pdf]

Supplementary Information:

UPRT, a suicide-gene therapy candidate in higher eukaryotes, is required for *Drosophila* larval growth and normal adult lifespan.

Arpan C. Ghosh[1], MaryJane Shimell[1], Emma R. Leof[1], Macy J. Haley[1], Michael B. O'Connor[1, *]

[1] Department of Genetics, Cell Biology and Development, University of Minnesota, Minneapolis, MN 55455, USA

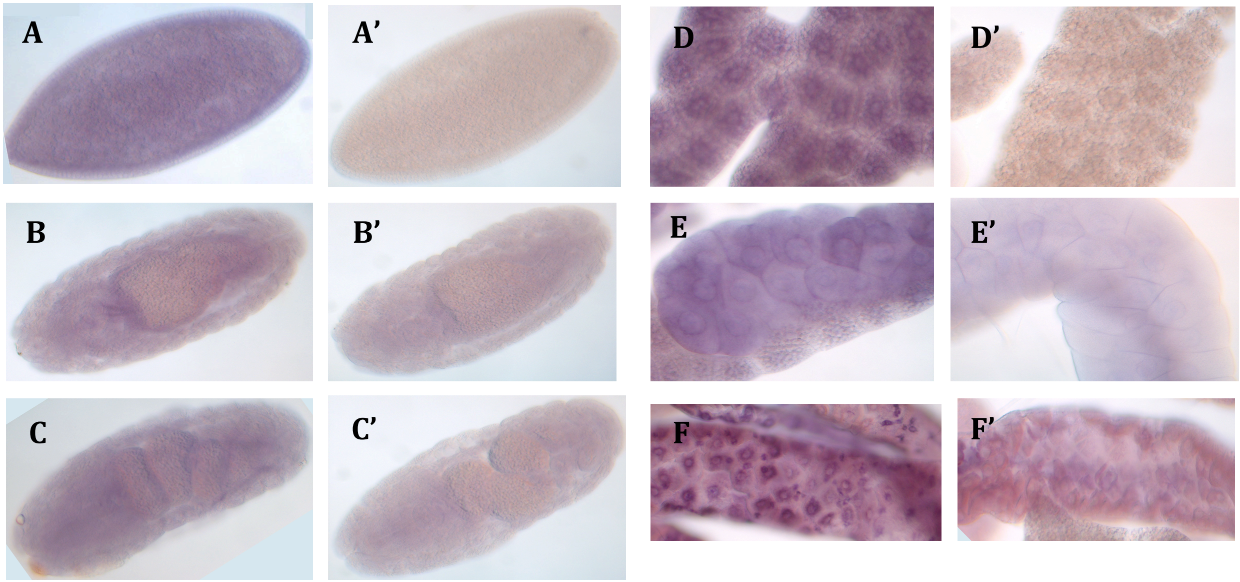

Figure S1: *kri in situ* hybridization. (A, A') embryonic expression at cellular blastoderm stage as revealed with antisense and sense probe respectively. (B, B'), germ band elongation stage. (C, C') stage 16 embryos (D, D'), Fat body from wondering third instar larva (E, E') Salivary gland from wondering third instar larva (F, F') hind gut from wondering third instar larvae.

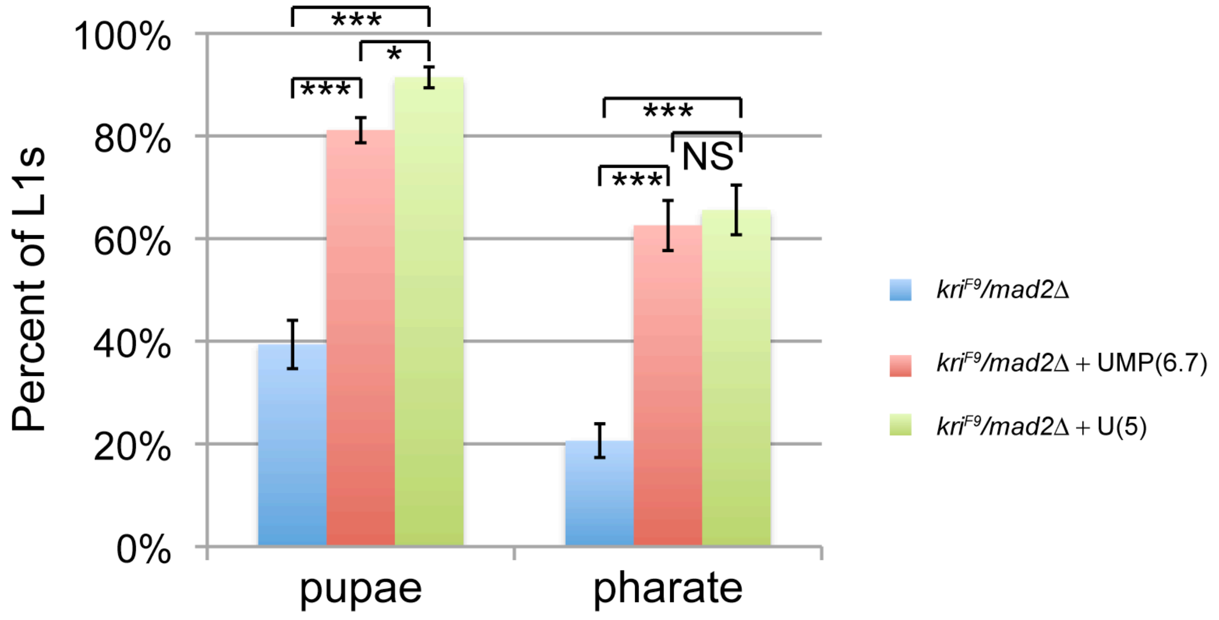

Figure S2: Comparison of *kri* mutant rescue by feeding equimolar amounts of UMP or Uridine. Larval and pupal viability of *kri*^{F9}*/mad2Δ* mutants reared on CMF with 6.7 mg/mL UMP, 5 mg/mL uridine, or no supplementation. *n* = 6 groups of 40 L1s each. All quantitative data presented as mean +/- SEM. p< .01 **, p<.001*** .

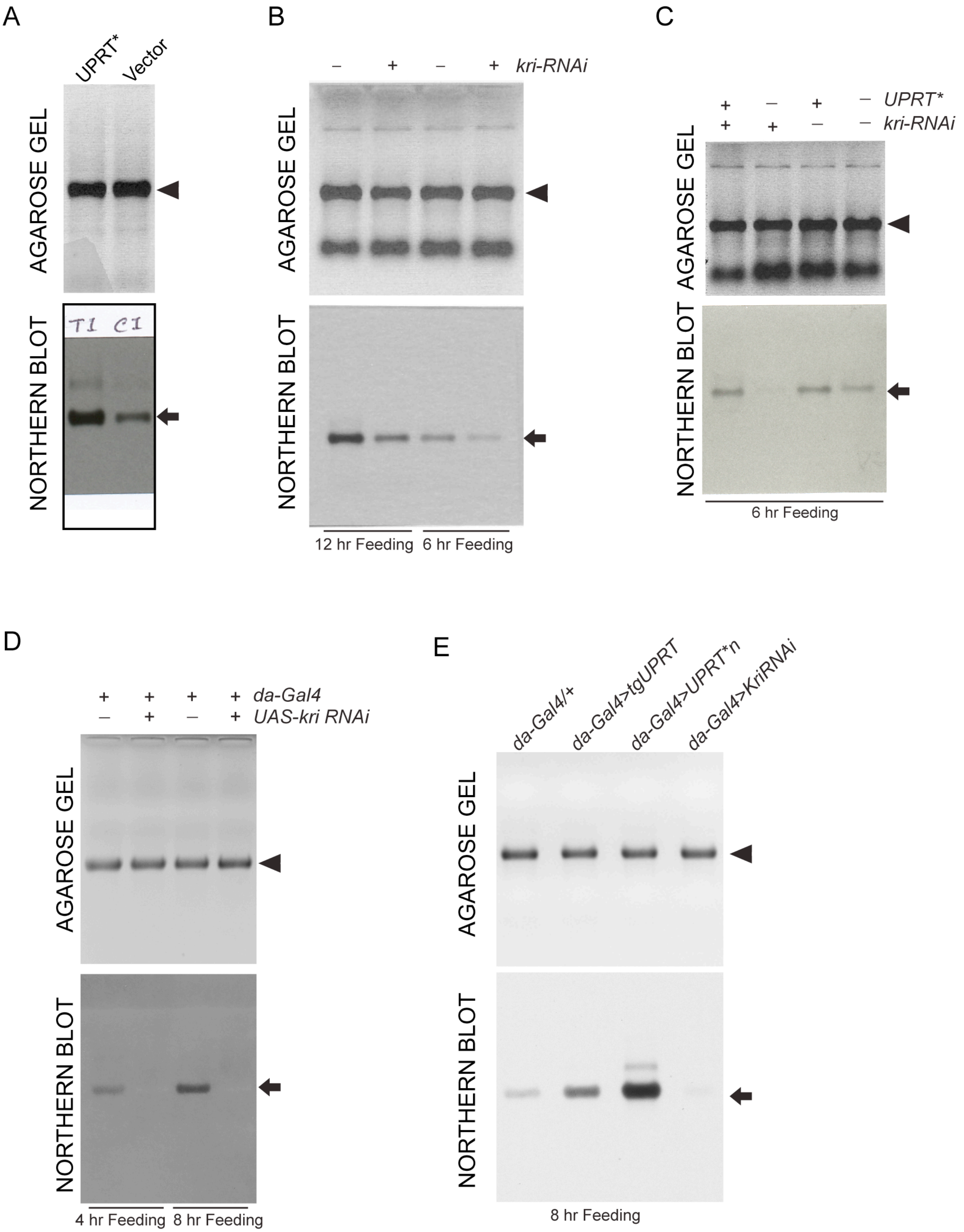

Figure S3: Shows full-length images of gels/blots shown in Figure 1, Figure 2 and Figure 5.

Arrow heads indicate the 26S RNA band on the EtBr stained agarose gel. Arrows indicate the same bad in the corresponding northern blot probing for thiolated 26S rRNA. (A) Full-length image of agarose gel and northern blot shown in Figure 1C. (B) Full-length image of agarose gel and northern blot shown in Figure 1E. (C) Full-length image of agarose gel and northern blot shown in Figure 1G. (D) Full-length image of agarose gel and northern blot shown in Figure 2A. (E) Full-length image of agarose gel and northern blot shown in Figure 5B. NB: The smaller RNA band in B and C was visible only in gels that were run for a shorter duration. This band never showed up in the northern blots presumably due to much lower abundance of newly incorporated 4-thioUracil.

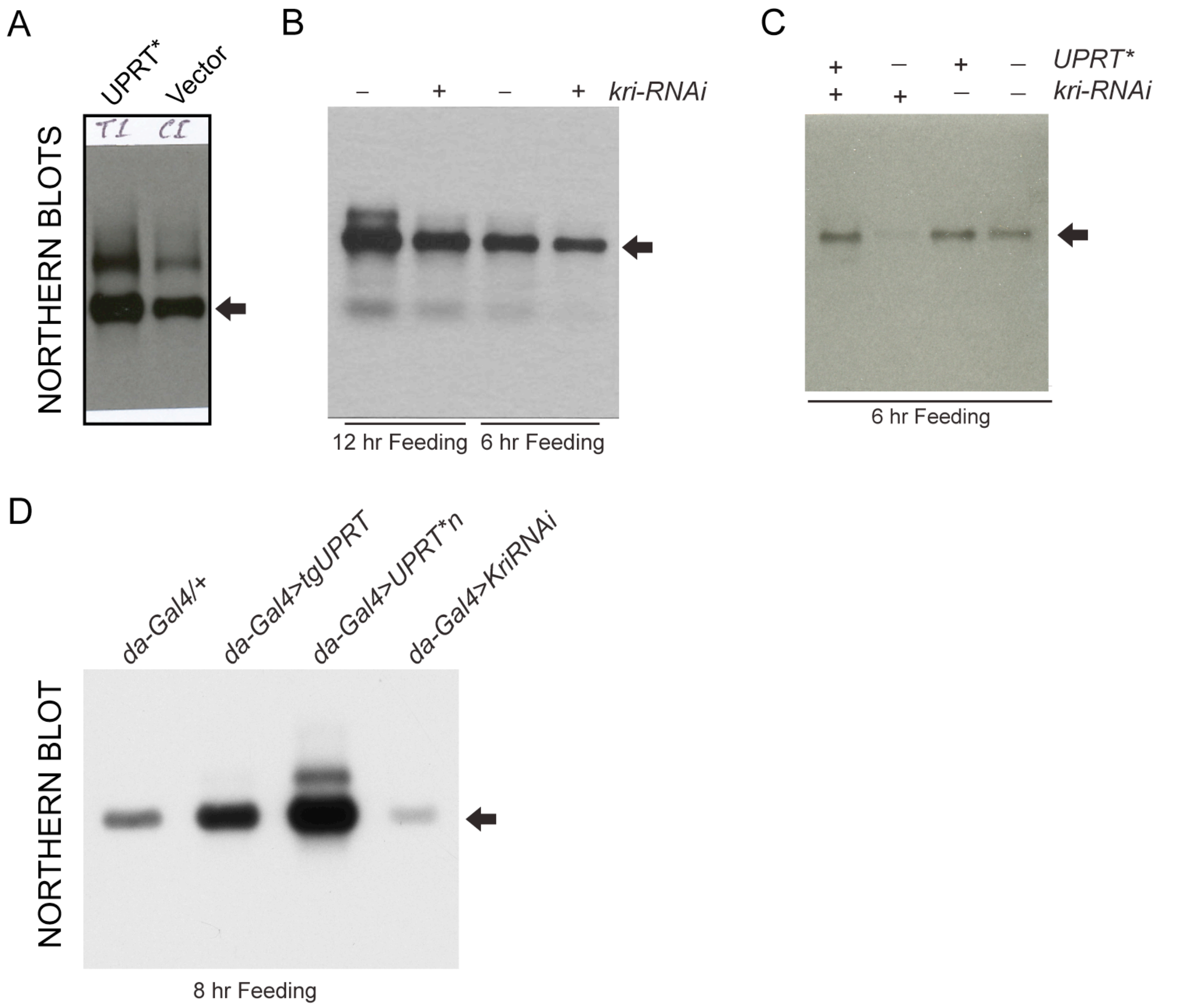

Figure S4: Shows longer exposures of northern blots shown in Figure 1 and Figure 5. Arrows indicate the thiolated 26S rRNA band. (A) 5 min exposure of blot shown in Figure 1C. (B) 5 min exposure of blot shown in Figure 1E. (C) 5 min exposure of blot shown in Figure 1G. (D) 30 seconds exposure of blot shown in Figure 5B.
